# R383C mutation of human CDC20 results in idiopathic non-obstructive azoospermia

**DOI:** 10.18632/oncotarget.21071

**Published:** 2017-09-16

**Authors:** Lingwei Li, Liqing Fan, Nanni Peng, Lihua Yang, Lisha Mou, Weiren Huang

**Affiliations:** ^1^ Institute of Human Reproduction and Stem Cell Engineering, Central South University, Changsha 410078, China; ^2^ Key Laboratory of Medical Reprogramming Technology, Shenzhen Second People’s Hospital, The First Affiliated Hospital of Shenzhen University, Guangdong 518035, China; ^3^ Reproductive Medical Center, People’s Hospital of Luohu District, Shenzhen, Guangdong 518001, China

**Keywords:** CDC20, CDC20 R383C mutant, Idiopathic azoospermia (IA)

## Abstract

Idiopathic azoospermia (IA) is a severe form of male infertility due to unknown causes. To investigate relative gene expression in human idiopathic non-obstructive azoospermia, we sequenced all the exons of cell division cycle 20 (CDC20) in 766 patients diagnosed with IA, as well as in 521 normally fertile men. Three novel missense mutations (S72G, R322Q, R383C) of CDC20 were detected and further confirmed by Sanger sequencing. The mRNA levels of securin, cyclin B, cyclin dependent kinase 1 (CDK1), and cyclin dependent kinase 2 (CDK2), which are all targeted for destruction via the anaphase-promoting complex/cyclosome^CDC20^ (APC/C^CDC20^) pathway, were detected at relatively high levels using real-time quantitative polymerase chain reaction analysis. This demonstrated that the CDC20 R383C mutation led to dysfunction during the transition from metaphase to anaphase and facilitation of mitotic exit *in vitro*, and caused prolonged mitotic arrest during the cell cycle. This study suggests that a CDC20 R383C mutation may result in the pathogenesis of human IA.

## INTRODUCTION

Approximately 15% of all couples suffer from fertility problems, and male infertility contributes to 30%-50% of these cases [1–5]. Genetic defects cause about 15%-30% of male infertility [4], with a quarter of those due to azoospermia. Obstructive disease makes up 30% of azoospermia, while the other 70% is idiopathic non-obstructive azoospermia (IA) [3–6].

Normal mitosis and differentiation can be influenced by genetic defects that are linked to physiological sperm function, which may result in spermatogenic obstacles and/or azoospermia. The causes of azoospermia can be multifactorial. Matzuk *et al* listed about 50 genes that are closely related to azoospermia, however CDC20 was excluded from the list [7]. CDC20 is a critical protein activation factor in the cell cycle, which was first confirmed in the S. cerevisiae’s cell reproductive cycle [8]. Human CDC20 homology, p55CDC, was first documented by Weinstein *et al* in 1994 [9]. The gene is located in human chromosome 1q34.1, and the nucleotide sequence includes 11 exons and 10 introns and codes 499 amino acids [9–10].

Anaphase-promoting complex/cyclosome (APC/C) is a multi-component ubiquitin ligase, which stimulates the progression of mitosis via proteolysis of several key cell cycle protein substrates. Genetic and biochemical investigations have shown that CDC20 can adhere to APC/C in mitosis, forming a large complex APC/C^CDC20^. The complex then activates the APC/C substrates, degrades securin and cyclin B via 26S proteasome, separates sister-chromatids, and promotes the normal transition from metaphase to anaphase. Besides securin and cyclin B, CDK1 and CDK2 should be also decreased before anaphase [8, 11–12].

Our recent work identified three novel missense mutations of four mutations in CDC20 from six IA patients with exome sequencing, which led us to investigate and evaluate the physiological effects of mutational forms of CDC20 in cell division.

## RESULTS

### Identification of CDC20 mutation in patients with IA

**Figure 1:**
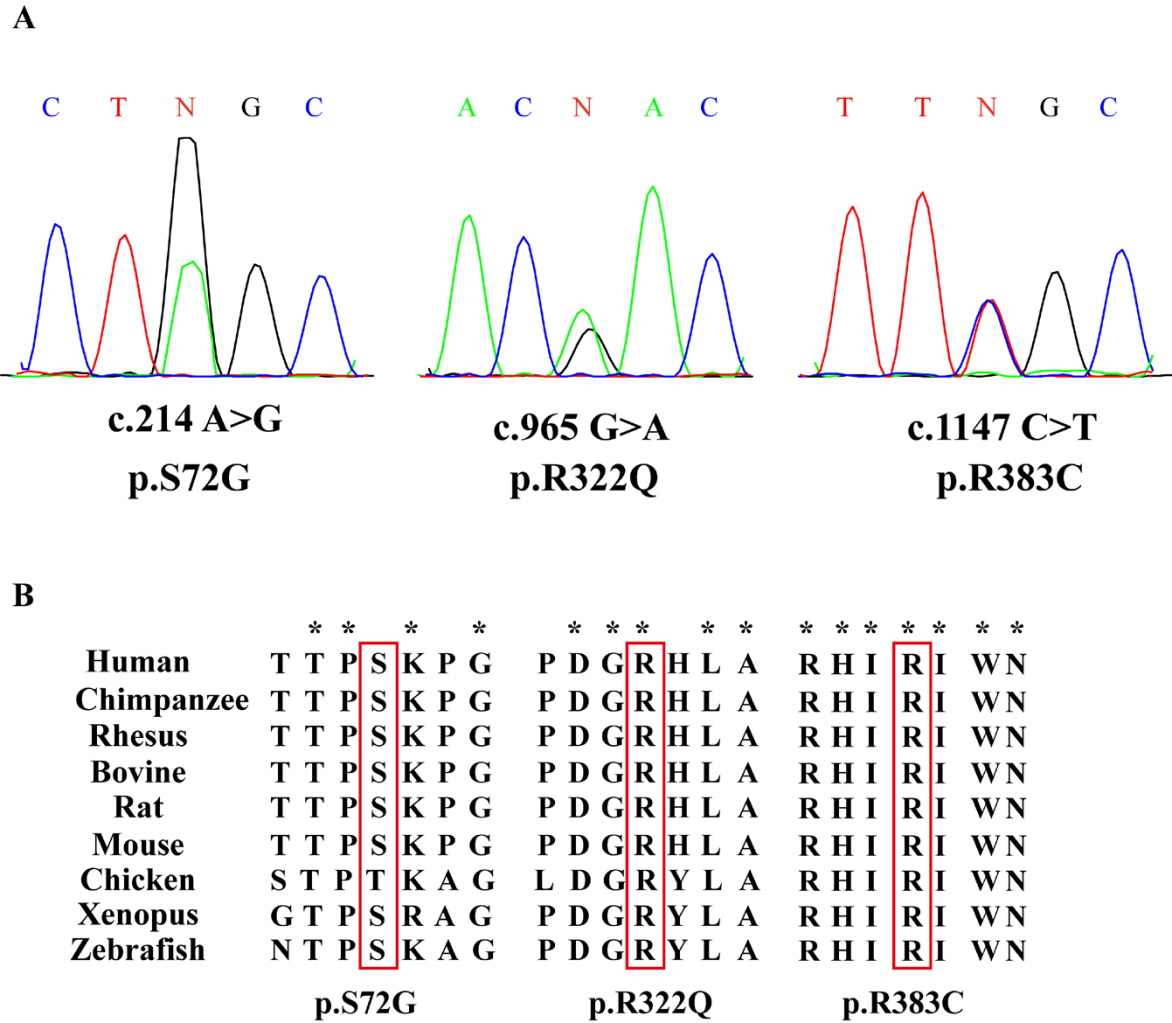
Three missense mutations of CDC20 identified in patients with IA **(A)** Chromatogram traces from Sanger sequencing showing the validated missense mutations. **(B)** Evolutionary conservation of amino acids affected by the missense mutations. Multiple protein alignments were performed with MegAlign (Demonstration System DNASTAR, Inc.). The identification numbers of CDC20 protein were as follows: human (NP_001246.2), chimpanzee (NP_001230901.1), rhesus (NP_001248046.1), bovine (NP_001075905.1), rat(NP_741990.1), mouse (NP_075712.2), chicken (NP_001006536.1), xenopus (NP_001079443.1), and zebrafish (NP_998245.2). The mutant alleles are boxed, and the star (^*^) shows the conserved residue.

To examine whether CDC20 genetic defects were associated with IA, we sequenced CDC20 exonic mutations in 766 IA patients and 521 normal men. Three novel CDC20 missense mutations were detected in six patients, and they were absent in the normal controls (Table 1). Three novel missense mutations had not been reported in either the dbSNP135 database or the 1000 Genome Project dataset. All these novel missense mutations were further confirmed by Sanger sequencing (Figure 1A). Alignment of the amino acid sequence of CDC20 to its orthologs in different species showed that the R383C mutation affected a highly conserved amino acid (Figure 1B).

**Table 1:** CDC20 mutations and SNPs identified in IA patients and fertile men

NO.	Sequence Variants	Amino acid changes	Patients (n=766)	Fertile men (n=521)
1	c.214 A>G	p.72 S>G	1	0
2	c.965 G>A	p.322R>Q	4	0
3	c.1147 C>T	p.383R>C	1	0

### Flow cytometry analysis of Hela and 293T cells transfected with CDC20 mutants

After the CDC20 R383C mutant was transfected into the cells, the G2/M cell ratio increased compared to those cells transfected with wild type (WT) or the other two CDC20 mutants (S72G or R322Q). Our data demonstrated that both the R383C mutant and the co-expression of R383C mutant and WT can cause prolonged cell arrest in the G2/M stage of the cell cycle (Figure 3, 4). This in turn leads to the lower ratio of G0/G1 cells (Figure 5). This suggested that the CDC20 R383C mutant could block cells from metaphase to anaphase.

**Figure 2:**
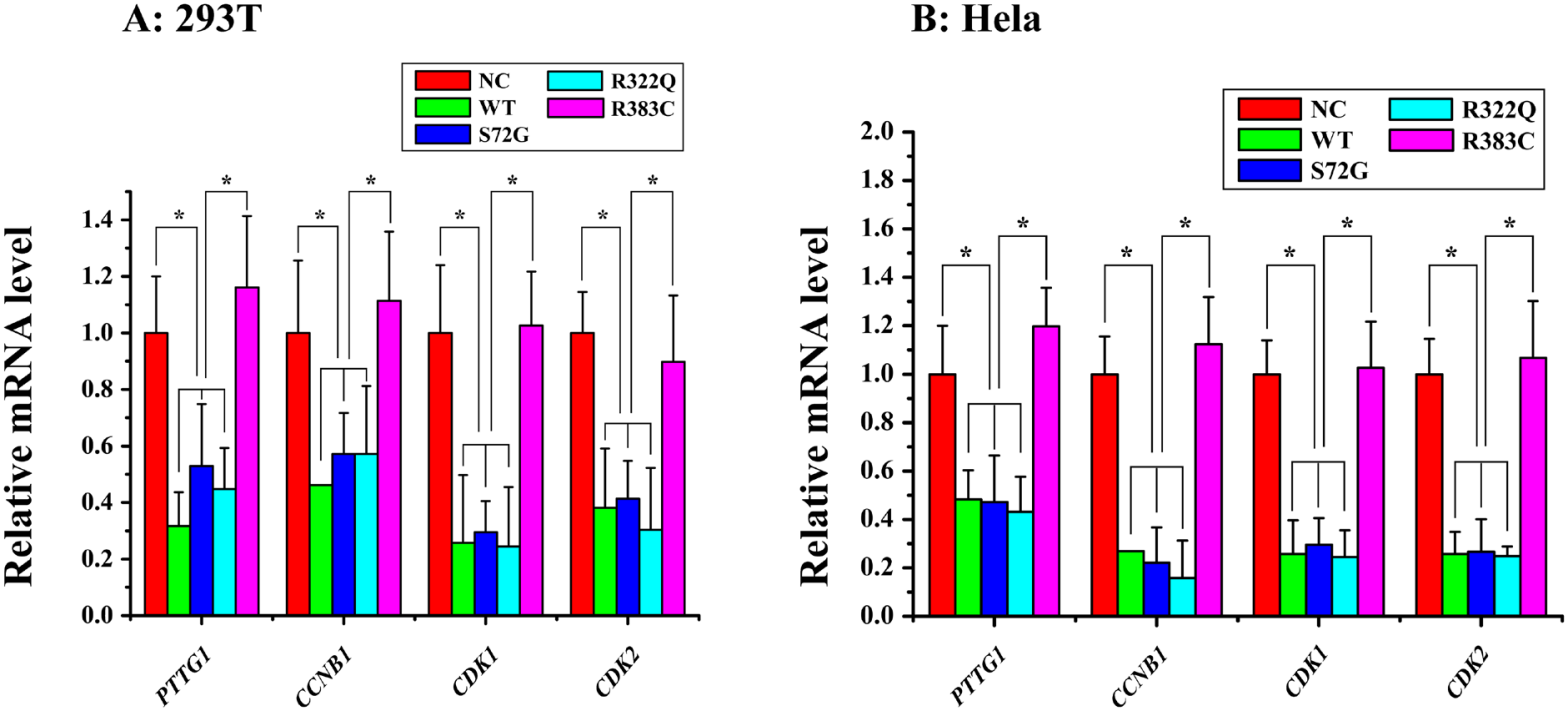
Relative mRNA expression of four proteins of interest in APC/C^CDC20 mutant^ pathway in HeLa and 293T cells Only in the CDC20 R383C mutant pathway, relative mRNA expression of all four kinds of regulators (PTTG1(securin), CCNB1(cyclin B), CDK1, CDK2) were increased compared with the CDC20 WT (^*^P<0.05).

**Figure 3:**
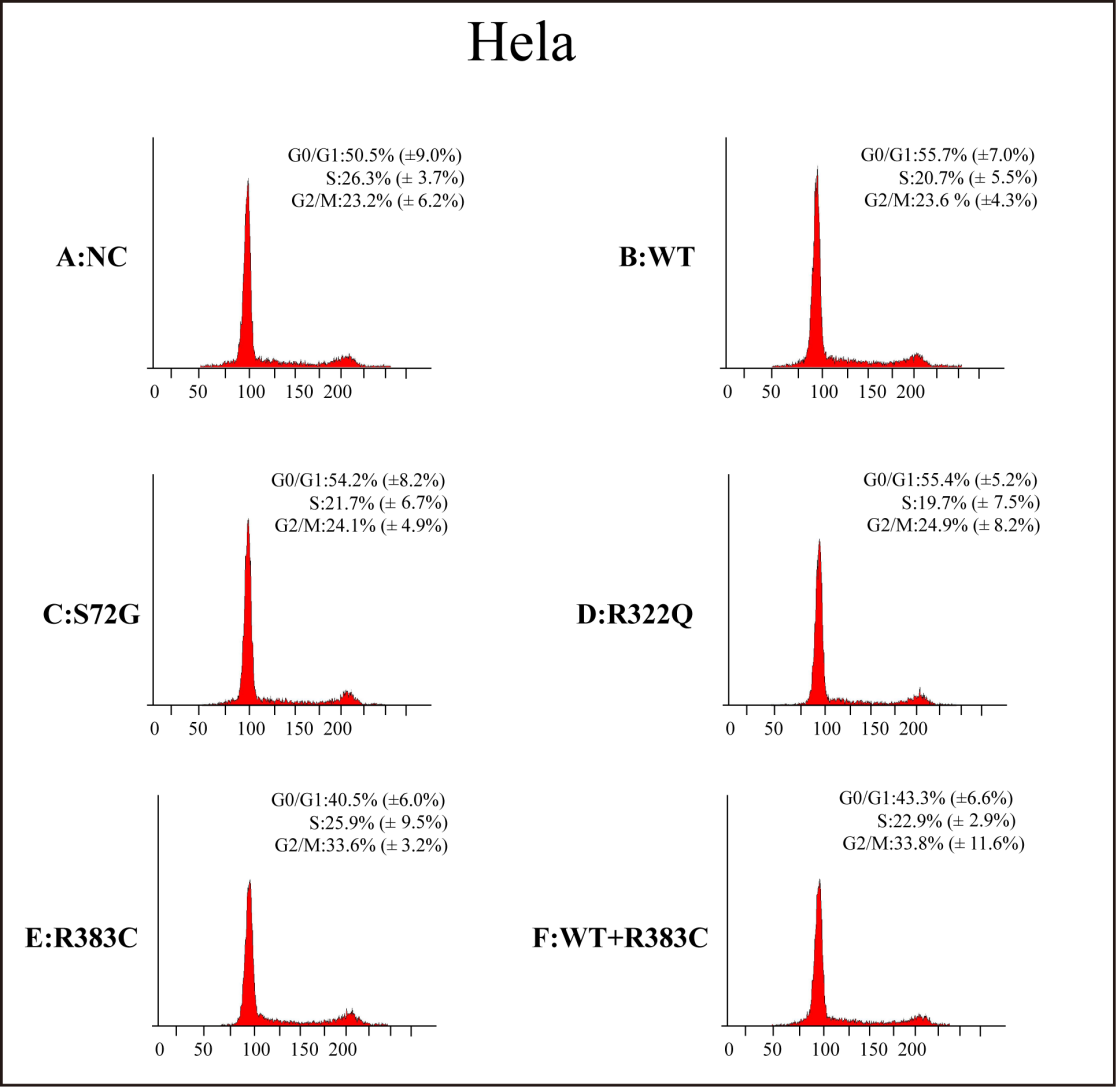
Flow cytometric analysis of HeLa cells transfected with the CDC20 mutant The relative high G2/M cell ratio indicates only those cells transfected with the CDC20 R383C mutant or co-transfected with the CDC20 R383C mutant and CDC20 WT were arrested in anaphase. WT: wild type, NC: no special control.

**Figure 4:**
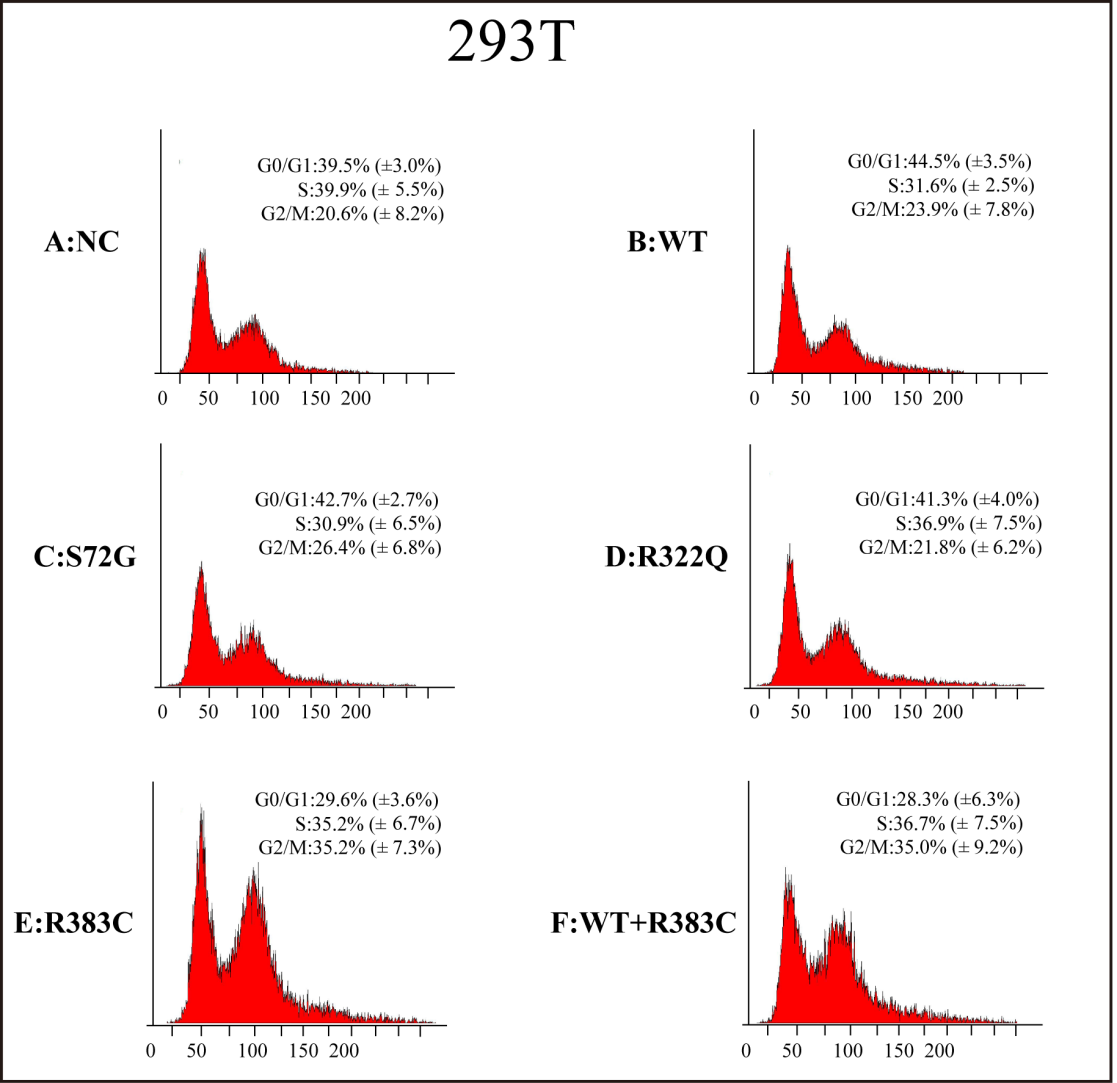
Flow cytometric analysis of 293T cells transfected with the CDC20 mutant The relative high G2/M cell ratio indicates only those cells transfected with the CDC20 R383C mutant or co-transfected with the CDC20 R383C mutant and CDC20 WT were arrested in anaphase. WT: wild type, NC: no special control.

**Figure 5:**
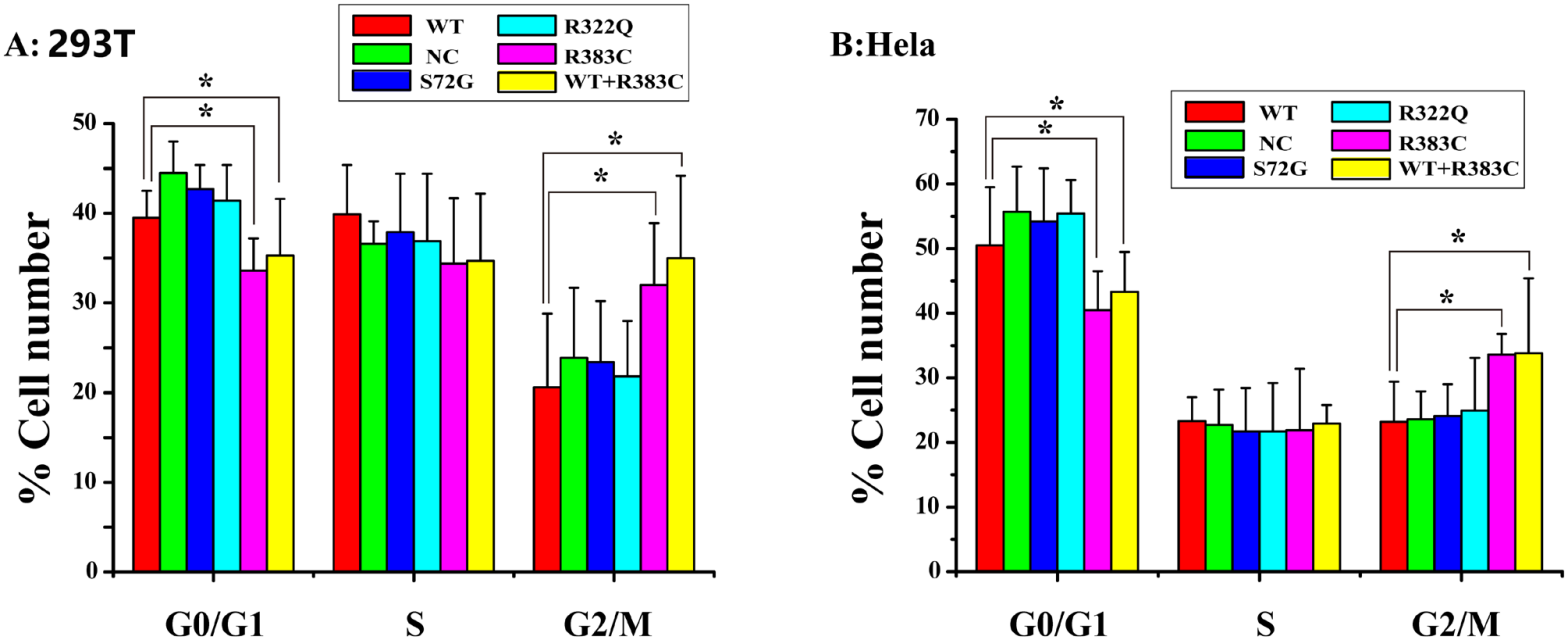
Cell count comparison in the cell cycle measured by flow cytometry in six cell lines between Hela and 293T cells Including wild type (WT) cells, no special control (NC) cells, cells were transfected three missense mutants (S72G, R322Q, R383C) and cells were co-transfected WT and R383C. Relative G2/M cell ratio increased when the cells were transfected with CDC20 R383C mutant, or co-transfected with CDC20 R383C mutant and CDC20 WT.

### Relative mRNA expression level of four regulators of APC/C^CDC20 mutant^ pathway

We detected the mRNA expression of securin, cyclin B, CDK1, and CDK2 after three CDC20 mutants were transfected into the cells. Our data indicated the levels of the four key proteins in APC/C^CDC20 R383C mutant^ pathway, which facilitate the progression of mitosis, were highly expressed when compared with WT or the other two CDC20 mutants (S72G, R322Q) (Figure 2). High securin and cyclin B mRNA expression may induce cell arrest.

### Dominant-negative effects of CDC20 R383C mutant

**Figure 6:**
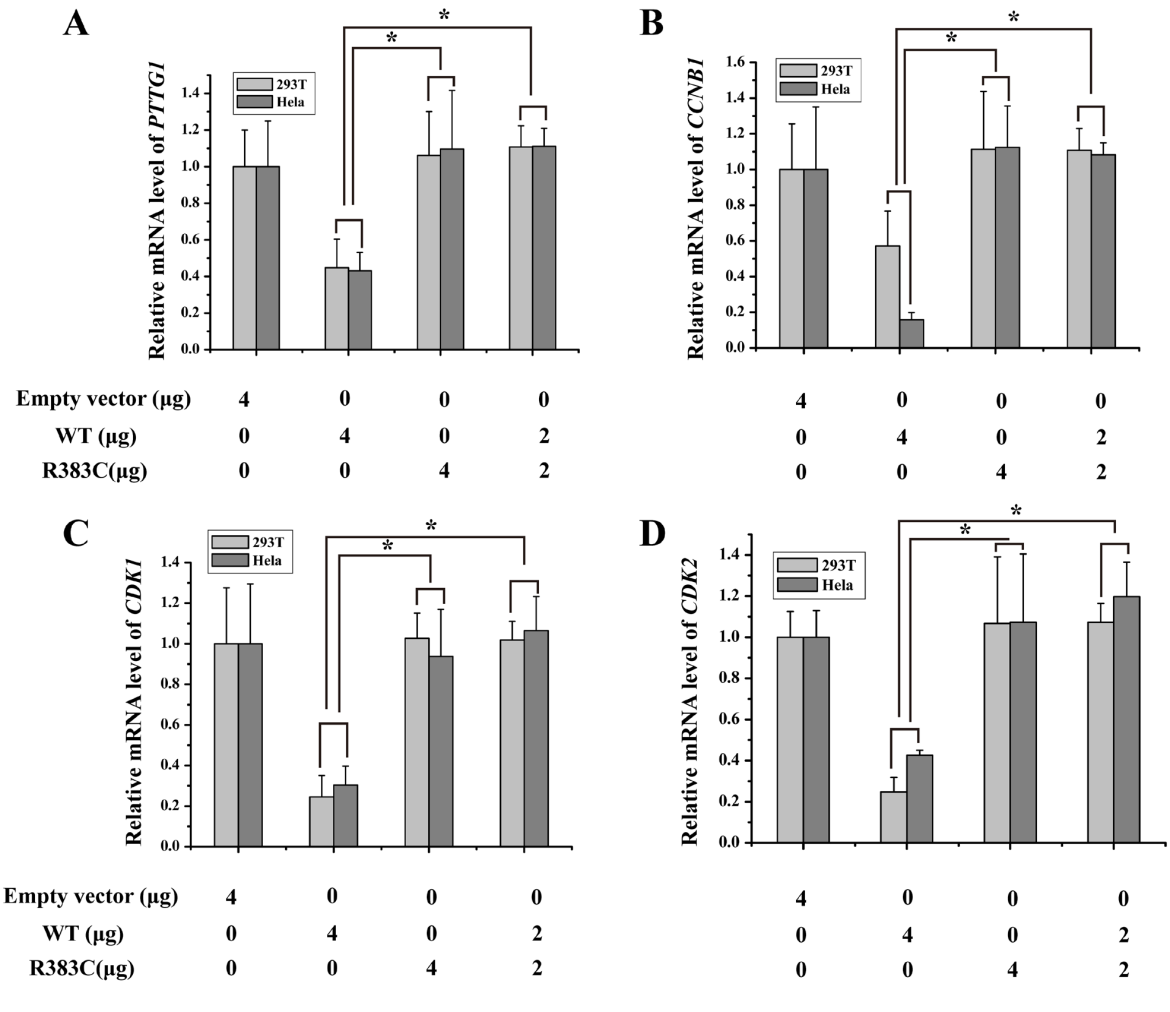
Histogram of four kinds of regulators of relative mRNA expression under dominant-negative effects of CDC20 R383C mutant HeLa and 293T cells were transfected with the CDC20 WT or CDC20 R383C mutant with the indicated doses. Levels of mRNA of four genes (PTTG1(securin), CCNB1(cyclin B), CDK1, CDK2) were increased when the cells were transfected with CDC20 R383C mutant compared with transfection of CDC20 WT (^*^P<0.05). The relative mRNA expression of four genes were detected by Real-Time PCR and compared to GAPDH. WT: wild type, NC: no special control.

To mimic patient heterozygosity, CDC20 R383C mutants and CDC20 WT were co-transfected into the cells. Again, the mRNA expression of four regulators increased after transfection of the CDC20 R383C mutant (Figure 6). These results indicated that the CDC20 R383C mutant can inhibit the transcriptional regulation activities of CDC20 WT through a dominant-negative effect. It can also suppress the degradation of other APC/C inhibitors, causing prolonged cell arrest.

## DISCUSSION

In this study, we identified three novel missense mutations of CDC20 in six IA patients. Among three mutations, only CDC20 R383C mutant was found in one of the 766 patients but absent in 521 fertile men. CDC20 R383C mutant resulted in heterozygous amino acid change at a conserved position. Arginine residue was replaced by cysteine residue in the R383C mutant, and the local alignment analysis of the amino acid sequences of CDC20 by MegAlign software suite suggests that the arginine residue was highly conserved in multiple vertebrates.

The evolutionary preservation of the entire region around this residue across multiple mammalian species indicated that mutations in this conservative region may have great influence on the CDC20 protein function. Our work indicates that the CDC20 R383C mutation can cause prolonged mitotic arrest in the cell cycle.

CDC20 promotes the destruction of securin and cyclin B that belong to the major substrates of APC/C^CDC20^, and trigger the separation of sister chromatids [8, 11–12]. During the cell cycle, the highest level of CDC20 protein occurs at the G2/M stage [13], which is enough to trigger the transition from metaphase to anaphase and maintain cellular division. Our study indicated that the ratio of those cells expressed with CDC20 R383C mutant and CDC20 R383C mutant co-transfected with CDC20 WT staying in G2/M phase was higher than other types of cells (Figure 5). With normal APC/C^CDC20^, securin and cyclin B should be degraded, and CDK1 and CDK2 should be inactivated [8]. Real-Time PCR analysis indicated the mRNA levels of securin, cyclin B, CDK1, and CDK2 remained high in cells with the CDC20 R383C mutant (Figure 2), which caused mitotic arrest in the cell cycle. Our results also demonstrated that the CDC20 R383C mutation not only led to a dysfunctional physiological effect during the cell cycle, but also had a dominant-negative effect on the WT allele, which may be the cause of the phenotypic effect of the heterozygous mutation in IA patients.

CDC20 dysfunction can cause prolonged mitotic arrest and apoptosis in cells, which suggests it could be a therapeutic target in cancer research [14]. Inactive CDC20 can cause infertility in male mice without hurting overall health and viability [15]. The molecular and cellular investigations confirmed that CDC20 R383C mutant could cause mitotic arrest in cell cycle. Our study also suggested that functional analysis of the genetic mutations may provide insights into a potential mechanism and feasible treatment of IA.

This study had a few limitations. As CDC20 is expressed in many tissues, it is unacceptable that a healthy individual with almost normal clinical characteristics except for IA carries CDC20 R383C mutant that can cause prolonged mitotic arrest and apoptosis in cells. The sample size of patients with the CDC20 R383C mutant is small, and individuals can reflect genetic heterogeneity. Secondly, it is well known that the gene expression presents continual spatio-temporal dynamics in the entire life span [16]. Because differential mitotic checkpoint mechanisms are required between the germ and somatic cells [17], the cellular pathogenic effect of the CDC20 R383C mutant might only affect germ cells, not somatic cells. Lastly, competition at the protein level may also cause a cell cycle delay. Despite these limitations, our results suggest that a CDC20 R383C mutation may result in idiopathic azoospermia.

## MATERIALS AND METHODS

### Sample preparation

From Jan 2007 to Oct 2011, DNA samples of 766 male patients with idiopathic azoospermia aged 24-46 years (average 30.6) were collected from the Center of Reproductive Medicine, Tongji Medical College, Huazhong University of Science & Technology. The samples that met the following criteria were included: (1) no sperm detected by microscope in the centrifugation pellets of semen samples at three different occasions, (2) no obstruction, inflammation, or injury of the reproductive system or pelvic cavity, (3) no endocrinological defect, and (4) no karyotypic abnormality and gene microdeletion in Y chromosome. Normal DNA samples of 521 men aged 29-51 years (average 39.6) were recruited from the Center of Physical Examination, Peking University Shenzhen Hospital as control. These normal people were proven fertility owing at least one child without any assisted reproductive techniques (ART) such as artificial insemination by donor (AID), *in vitro* fertilization (IVF), or intracytoplasmic sperm injection (ICSI). All the sample collection and experiments were approved by the Ethics Committee of Tongji Medical College, Huazhong University of Science & Technology and Peking University Shenzhen Hospital. Informed written consent was obtained from all patients [18]. A large scale and parallel exome sequencing including candidate genes linked to male reproductive system were detected in the two groups. Dozens of target genes were screened from the IA patients according to the relative known gene data bank [18].

### Validation of missense mutations

Sample preparation and exome sequencing have been or will be accomplished and published elsewhere [18]. To validate the three novel missense mutations of CDC20, Sanger sequencing was performed by 3730 DNA analyzer (Applied Biosystems, Carlsbad, CA, USA). The corresponding primer sequences for PCR and Sanger sequencing were listed in Table 2.

**Table 2:** Primers used for PCR and Sanger sequencing validation of *CDC20* gene

Exon	Primer ID	Sequence (5'-3')	Product Length
2	CDC20-mut1-F	CCACAGCGCCGGCAGGACTC	461bp
	CDC20-mut1-R	CCCTCTGGCGCATTTTGTGGTTTT	
7	CDC20-mut2-F	TATTTGCCCACCCTCCCCTTGACT	550bp
	CDC20-mut2-R	ACTGGTGCCCCCTCCTGTTGC	
8	CDC20-mut3-F	CTGGGGACTGTTAAGGGGAGAAGG	465bp
	CDC20-mut3-R	GAGGCAAGGGGAAGTGACAAGGTT	

### Construction of Plasmids via site-directed mutagenesis

Three novel validated missense mutations (S72G, R322Q, R383C) of CDC20 were generated by site-directed mutagenesis in the full length cDNA sequence, as inserted into pcDNA3.1+ (Invitrogen, Carlsbad, CA, USA) and co-expressed with plasmids respectively. The induced mutation sequences were verified by Sanger sequencing. Primers used for site-directed mutagenesis construction are shown in Table 3.

**Table 3:** Primers used for site-directed mutagenesis construction

Primer ID	Sequences (5'-3')
CDC20-mut1-F(S72G)	GGTTCAGACCACTCCTGGCAAACCTGGCGGTGA
CDC20-mut1-R(S72G)	TCACCGCCAGGTTTGCCAGGAGTGGTCTGAACC
CDC20-mut2-F(R322Q)	TGGGCCCCAGATGGACAACATTTGGCCAGTGGT
CDC20-mut2-R(R322Q)	ACCACTGGCCAAATGTTGTCCATCTGGGGCCCA
CDC20-mut3-F(R383C)	AGTGATCGACACATTTGCATCTGGAATGTGTGC
CDC20-mut3-R(R383C)	GCACACATTCCAGATGCAAATGTGTCGATCACT

### Cell culture, transfection, and flow cytometry

HeLa cells and 293T cells (ATCC, Manassas, VA, USA) were cultured in Dulbecco’s Modified Eagle’s Medium (Gibco BRL, Gaithersburg, MD, USA) supplemented with 10% (v/v) fetal bovine serum (FBS). They were incubated at 37°C in a 5% CO_2_ humidified incubator, and seeded in 6-well tissue culture plates 24 h prior to transfection. Transfection was performed using Lipofectaminen 2000 (Invitrogen, Carlsbad, CA, USA) according to the supplier's instructions. Equivalent amounts (4 μg) of pcDNA3.1+CDC20 plasmid expression system (WT and mutant) or pcDNA3.1+ empty vector were added into one well consisting of 4 ×10^6^ million cells, including 5 μL Lipofectaminen 2000 solution. Cells were harvested 48 h after transfection and then were used for DNA content analysis by flow cytometry (Beckman, Brea, CA, USA).

Cells were re-suspended in cold PBS after centrifugation at 1200 g for 5 min, then the supernatant was discarded, and the cells were fixed by drop-wise addition of cold 100% ethanol with gentle vortex. After fixation, the cells were re-suspended in 70% ethanol (30% PBS) and stored at 4 °C overnight, washed again with PBS, digested in RNase A (Takara, Dalian, Japan) (50 μg/ml) solution for 30 min in 37°C water bath, and stained with propidium iodide (Invitrogen, Carlsbad, CA, USA) (50 μg/ml). Samples of 10,000 cells were analyzed by the Cell Quest software (Becton–Dickinson, Hercules, CA, USA).

### RNA extraction and real-time PCR

Total RNAs were extracted from HeLa cells and 293T cells using the TRIzol (Invitrogen, Carlsbad, CA, USA) according to the manufacturer’s instructions. The first-strand cDNA was synthesized using oligo-dT primers (K1622, Fermentas, Waltham, MA, USA). Primers used for Real-time PCR were listed in Table 4. The PCR was performed at 98 °C for 2 min, 30 cycles of 98 °C for 10 s, 55 °C for 30 s, and 72 °C for 30 s, with a final 72 °C for 5 min. Real-time PCR was performed using the SYBR^®^ Premix EX Taq™ II PCR Kit (RR820A, Takara, Dalian, Japan) according to the manufacturer’s instructions on the Roche Light-cycler 480 Real-Time PCR System (Roche, Rotkreuz, Switzerland). Data were calculated according to the Applied Biosystems comparative Ct method.

**Table 4:** Primers used for RT-PCR

Primer ID	Sequence (5'-3')	Product Length
PTTG1 (securin)	CGCGGGTGGTTAGTTGAGCGTTGGTCCAGTTAGCAGTC	154bp
CCNB1 (cyclinB)	GGACACCAACTCTACAACATTACCCGATGTGGCATACTTGTTCTTGA	140bp
CDK1	CGCAACAGGGAAGAACAGTAAGCCAAGATAAGCAACTCCTT	132bp
CDK2	ACCAGTGTAGTAATGAGCAGAAGCTATGCCTGATTACAAGCCAAGTT	89bp

### Statistical analysis

All experiments were repeated at least three times. Statistical analysis was performed using GraphPad Prism Version 5.0 (GraphPad Software, San Diego, CA, USA). Values were expressed as mean ± SD, with P value < 0.05 considered statistically significant.
